# Ex Vivo Line-Field Confocal Optical Coherence Tomography and Ex Vivo Fusion Confocal Microscopy for Lateral-Margin Assessment of Basal Cell Carcinoma in Correlation with Histopathology: A Prospective Pilot Diagnostic-Accuracy Study

**DOI:** 10.3390/cancers18152509

**Published:** 2026-08-05

**Authors:** Kristina Fünfer, Marco Mozaffari, Hanna Illium, Sophia Schlingmann, Oliver Mayer, Maximillian Deußing, Elke Sattler, Julia Welzel, Sandra Schuh

**Affiliations:** 1 Department of Dermatology and Allergology, University Hospital Augsburg, 86179 Augsburg, Germany, sandra.schuh@uk-augsburg.de (S.S.); 2 Department of Dermatology and Allergology, LMU University Hospital, 80337 Munich, Germany

**Keywords:** basal cell carcinoma, line-field confocal optical coherence tomography, ex vivo imaging, margin assessment, histology, Mohs micrographic surgery, ex vivo fusion confocal microscopy, one-stop-shop

## Abstract

**Background/Objectives**: Rapid optical assessment of freshly excised basal cell carcinoma (BCC) specimens may shorten the interval between excision and margin evaluation. This prospective pilot study estimated the diagnostic performance of ex vivo line-field confocal optical coherence tomography (LC-OCT) for detecting residual BCC at lateral surgical margins, using histopathology as the reference standard. **Methods**: Fifty-five clinically suspicious lesions from 48 patients were examined at two centers. Results were analyzed for the complete cohort and for a post-training inclusion phase comprising 32 lesions from 31 patients. Overall lesion-level consensus classifications were recorded separately from classifications of the four clock-face quadrants. Lesions with a negative overall LC-OCT assessment and at least one non-evaluable quadrant were classified as indeterminate and excluded from the primary lesion-level analysis. Thirteen lesions also underwent exploratory ex vivo fusion confocal microscopy (evFCM). **Results**: In the inclusion phase, 125 of 128 quadrants were evaluable. Thirteen true-positive, 106 true-negative, 2 false-negative, and 4 false-positive quadrant results yielded 86.7% sensitivity, 96.4% specificity, and 95.2% accuracy. At the lesion level, 2 of 32 results were indeterminate. Among the remaining 30 lesions, LC-OCT yielded 5 true-positive, 22 true-negative, 2 false-negative, and 1 false-positive results, corresponding to 71.4% sensitivity, 95.7% specificity, and 90.0% accuracy. The evFCM analysis was descriptive because the subset was small and nonconsecutive. **Conclusions**: Ex vivo LC-OCT showed promising specificity and overall accuracy for assessing lateral BCC margins after training. The limited number of margin-positive lesions, indeterminate results, clustered quadrant data, and absence of systematic deep-margin imaging require cautious interpretation. LC-OCT should not replace histopathological margin assessment on the basis of the present data.

## Simple Summary

Basal cell carcinoma is commonly treated by surgical excision. When margin-controlled surgery is performed using paraffin-embedded sections, the wound may remain open until histopathological results are available. This prospective pilot study evaluated whether ex vivo line-field confocal optical coherence tomography (LC-OCT) can identify residual basal cell carcinoma at the lateral margins of freshly excised specimens. Fifty-five lesions were examined. The primary post-training phase comprised 32 lesions; two had indeterminate lesion-level LC-OCT results, leaving 30 lesions for the primary lesion-level analysis. Compared with histopathology, LC-OCT achieved 90.0% lesion-level accuracy and 95.2% quadrant-level accuracy in the post-training phase. However, only seven lesions had histologically positive lateral margins, resulting in substantial uncertainty around sensitivity. Deep margins were not assessed systematically. Ex vivo LC-OCT may support rapid lateral-margin assessment, but histopathological verification remains required, and larger studies with standardized imaging, prospectively documented reader assessments, and systematic deep-margin evaluation are needed before routine clinical implementation.

## 1. Introduction

Optimizing medical care is a major contemporary challenge. As the population grows, healthcare systems must operate efficiently to meet increasing demand. Basal cell carcinoma (BCC), which affects a steadily rising number of individuals, illustrates these challenges [1]. Depending on the subtype, location, and depth of tumor invasion, margin-controlled surgery is often the treatment of choice [1]. In (slow) Mohs micrographic surgery using paraffin-embedded sections, the tumor is excised, and histopathological evaluation is required before determining whether further resection is necessary or the wound can be closed [2]. This process may require multiple surgical sessions, burden patients, and increase personnel and material costs [2]. Even when no further excision is needed, patients may remain hospitalized for inpatient management of an open wound while awaiting histopathological results. Histopathology remains the reference standard for surgical margin assessment and is expected to retain this role. Nevertheless, in settings where rapid histopathological examination is unavailable, complementary diagnostic strategies, such as advanced dermatological imaging techniques, may help accelerate margin assessment and improve surgical workflows [3]. Line-field confocal optical coherence tomography (LC-OCT), which combines optical coherence tomography and reflectance confocal microscopy, provides high-resolution vertical and horizontal imaging of the skin [4]. It has shown promising results for the in vivo diagnosis and characterization of BCC [5]. Originally developed for in vivo use, LC-OCT can also be applied to freshly excised specimens, similar to ex vivo fusion confocal microscopy (evFCM), following recent technical developments [6].

Ex vivo fusion confocal microscopy (evFCM) is another imaging modality that enables rapid microscopic evaluation of freshly excised tissue and can generate images resembling conventional hematoxylin and eosin staining [7]. Although evFCM has already been investigated for intraoperative margin assessment, the role of ex vivo LC-OCT in BCC margin evaluation remains insufficiently explored [8]. A previous methodological study from our group introduced the “BCC-One-Stop-Shop” concept and described the technical co-localization system, AI-supported visualization, and standardized in vivo and ex vivo LC-OCT workflows [9]. That publication illustrated the proposed workflow using two representative clinical cases and did not report cohort-level diagnostic-performance analyses. It explicitly indicated that the diagnostic evaluation of the larger prospective ex vivo cohort would be presented separately. The present study constitutes this previously announced diagnostic-accuracy analysis and focuses specifically on the histopathology-referenced performance of ex vivo LC-OCT for surgical margin assessment.

The aim of this prospective two-center study was therefore to evaluate the diagnostic performance of ex vivo LC-OCT for detecting residual BCC at the surgical margins of freshly excised specimens, using conventional histopathology from slow Mohs micrographic surgery as the reference standard. Diagnostic performance was assessed at both the lesion and quadrant levels. In selected cases, an exploratory descriptive comparison with evFCM was also performed.

## 2. Materials and Methods

**Figure 1:**
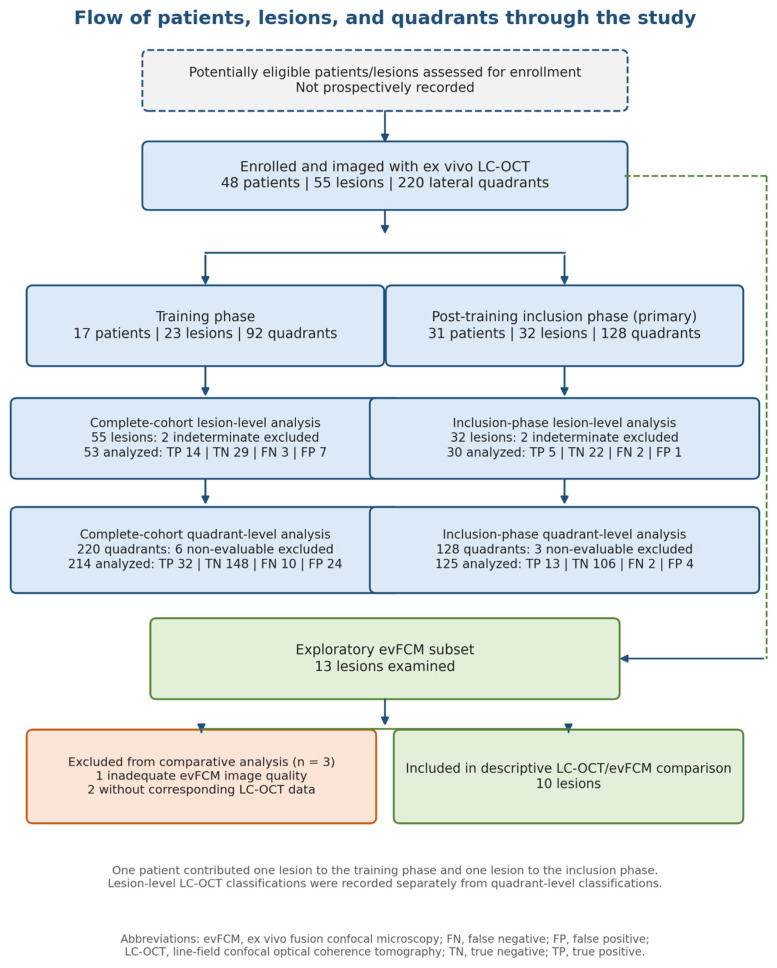
Flow of patients, lesions, and quadrants through the study. The number of potentially eligible patients or lesions assessed but not enrolled was not prospectively recorded. One patient contributed one lesion to the training phase and one lesion to the post-training inclusion phase. Lesion-level LC-OCT classifications were recorded separately from quadrant-level classifications. evFCM, ex vivo fusion confocal microscopy; LC-OCT, line-field confocal optical coherence tomography.

This prospective, two-center pilot diagnostic-accuracy study was conducted at University Hospital Augsburg and LMU University Hospital Munich. The relevant protocols were approved by the Ethics Committee of Ludwig Maximilian University of Munich before enrollment in the applicable study phases (protocol 22-0781 for the use and training of artificial intelligence, approved 11 November 2022; protocol 22-0922 for postoperative ex vivo LC-OCT margin assessment, approved 22 December 2022), and the study was conducted in accordance with the Declaration of Helsinki. The study is reported in accordance with the Standards for Reporting Diagnostic Accuracy Studies (STARD 2015 [10]). The completed checklist is provided as Supplementary Table S1, and the flow of patients, lesions and quadrants through the study is shown in Figure 1.

### 2.1. Patients

The study included adults aged 18 years or older with lesions clinically suspicious for BCC who were scheduled for surgical excision at one of the two participating centers. Eligible lesions constituted a convenience sample and were not enrolled consecutively. Written informed consent was obtained from all participants. The clinical suspicion of BCC was either confirmed by a previous biopsy or supported by dermoscopy and OCT or LC-OCT examination.

Lesions larger than 6 cm^2^ were excluded because they exceeded the capacity of the available sample tray. Additional exclusion criteria comprised insufficient overall image quality or incomplete imaging that prevented the planned assessment. In total, 55 lesions were imaged between December 2022 and December 2024, including 11 lesions from Munich and 44 from Augsburg. The total number of potentially eligible patients or lesions assessed but not enrolled was not prospectively recorded and could not be reconstructed.

Because ex vivo LC-OCT is a relatively new technique, an initial training phase was used to establish familiarity with the imaging modality and study protocol. Before outcome analysis, examinations were assigned to either the training phase or the post-training inclusion phase. The planned training phase ended on 30^th^ September 2023 after the supervised examinations had been completed. Three lesions, acquired after that date, were nevertheless assigned to the training phase because they represented each examiners’ first measurement. The primary diagnostic-accuracy analysis was performed for the post-training inclusion phase (October 2023 to December 2024), and the complete cohort was analyzed secondarily to explore potential learning effects.

In addition, 13 cases were examined using evFCM for an exploratory descriptive comparison.

The present study is part of the same research program as the previously published methodological description of the “BCC-One-Stop-Shop” workflow [9]. That publication presented two representative cases to illustrate the technical co-localization system, AI-supported visualization, and standardized in vivo and ex vivo imaging procedures, but did not report cohort-level diagnostic-performance results. The current manuscript presents the previously announced prospective diagnostic-accuracy analysis of the ex vivo cohort and provides blinded, histopathology-referenced lesion- and quadrant-level results.

### 2.2. Line-Field Confocal Optical Coherence Tomography

For this study, the line-field confocal optical coherence tomography (LC-OCT) device deepLive^TM^ developed by DAMAE Medical (Paris, France) was used. This imaging system combines optical coherence tomography (OCT) with reflectance confocal microscopy (RCM). A line of light is projected onto the tissue, and the backscattered signal is captured by a line-scan camera. An integrated algorithm then processes the signal by detecting interference in the amplitude to generate high-resolution images [4].

The device captures real-time cellular-level images at a rate of 8 frames per second, with a lateral resolution of approximately 1 µm and a penetration depth of approximately 500 µm [11].

**Figure 2:**
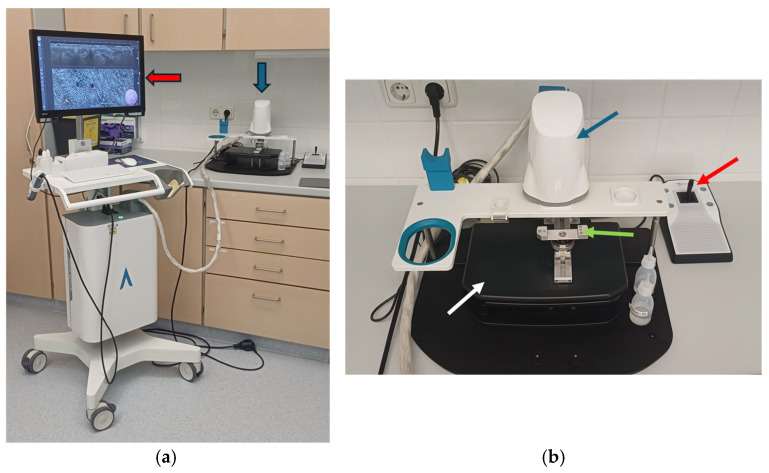
(**a**) deepLive^TM^ Line-field confocal optical coherence tomography (LC-OCT) system (DAMAE Medical, Paris, France) configured for ex vivo examination. The blue arrow indicates the ex vivo platform, while the red arrow shows the device with the software operating in ex vivo mode. (**b**) Ex vivo LC-OCT setup. The blue arrow shows the handheld probe mounted on the ex vivo platform. The red arrow indicates the joystick used to control the motorized stage, the white arrow shows the motorized plate. The green arrow indicates the magnetic mount for the sample holder.

To use the device in the ex vivo mode, the in vivo handheld unit was mounted onto a specially designed ex vivo imaging platform (Figure 2). This required removal of the glass window and replacement with a customized holder, allowing the handheld device to be securely positioned. The tissue sample was placed in a specially designed sample holder with a drop of paraffin oil to improve optical coupling. Silicone oil was then instilled between the handheld unit and the sample holder glass as an immersion medium.

The sample holder was mounted on a motorized plate, allowing precise lateral movement. The tissue could also be moved manually toward the LC-OCT handheld unit in the horizontal plane. In ex vivo mode, several imaging options are available. As in the in vivo mode, the system provides two-dimensional horizontal imaging with a field of view (FOV) of 1.2 mm × 0.5 mm, two-dimensional vertical imaging with a FOV of 1.2 mm × 0.4 mm, and three-dimensional imaging with an image size of 1.2 mm × 0.5 mm × 0.4 mm.

Simultaneously, a color image of the scanned tissue surface with a diameter of 2.5 mm is displayed on the screen to aid with orientation. Both vertical and horizontal real-time imaging modes are available, with the motorized plate movement allowing dynamic scanning. Images and videos can be recorded during examination. The tissue can also be scanned using a 3D mosaic mode, which extends the FOV. In a study by Ogien et al., this mode was used to image two tissue samples, achieving extended FOVs of 3.4 mm × 2.6 mm × 0.3 mm and 3 mm × 3 mm × 0.25 mm, respectively [6].

Additionally, the system includes an artificial intelligence (AI)-generated BCC probability score, which displays the likelihood of BCC as a percentage. This feature has already been established for in vivo application [9,12].

### 2.3. Ex Vivo Fusion Confocal Microscope (evFCM)

The VivaScope^®^ 2500M-G4 (VivaScope GmbH, Munich, Germany) was used for evFCM imaging in this study. This reflectance confocal microscope uses two lasers with wavelengths of 488 nm and 785 nm. The laser light is projected onto a defined region of the tissue, and the reflected signal is then measured. A gating pinhole ensures that only the light reflected from the focal plane is captured, allowing precise imaging of cellular structures.

The resulting images are rendered in different shades of gray depending on the refractive indices of the cellular components. To enhance the visualization of certain structures, tissue samples can also be stained with the fluorescent dye acridine orange, which improves contrast and detail. The acquired images can then be digitally processed and merged using a specialized algorithm to generate virtual sections resembling hematoxylin and eosin (H&E) staining.

The device offers a horizontal resolution of less than 1.25 µm, a vertical resolution of less than 5.0 µm, and a penetration depth of up to 200 µm. The maximum field of view is 550 µm × 550 µm, and the images can be magnified up to 550× [8,13].

### 2.4. Study Protocol

The procedure was integrated into the standard slow Mohs micrographic surgery workflow using paraffin-embedded sections at both centers. Patients were treated according to the routine clinical protocol. Tumor margins were identified by the surgeon through visual inspection and dermoscopy, with a standard safety margin of 2–3 mm. The lesions were then excised by dermatologic surgeons and the specimens were stored in isotonic saline (NaCl) solution to preserve tissue integrity for immediate imaging.

The specimen was first imaged using the LC-OCT device. For this purpose, the handheld LC-OCT unit was adapted for ex vivo use by activating the corresponding software mode and replacing the lens attachment with a customized ex vivo adapter. The handheld device was then mounted onto the dedicated ex vivo imaging platform (Figure 2).

**Figure 3:**
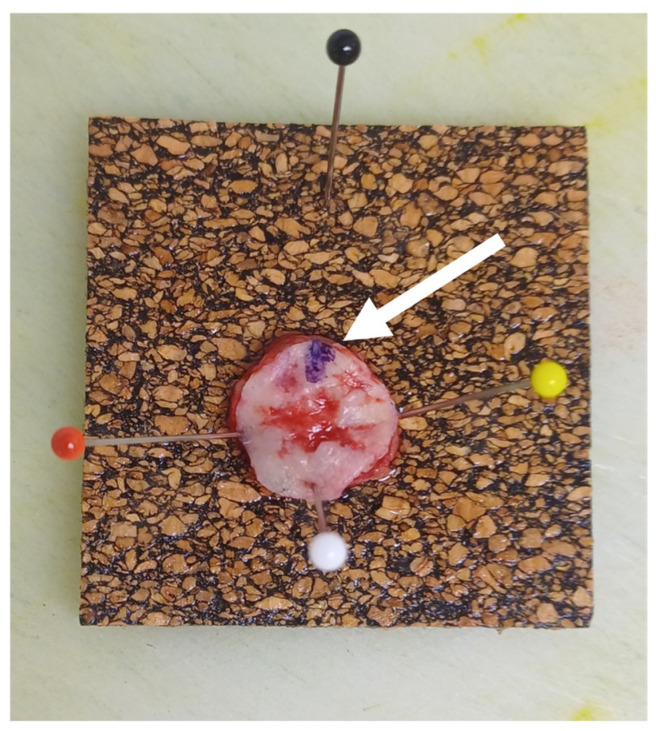
Excised basal cell carcinoma (BCC) specimen marked with color-coded needles for orientation: black indicates the cranial aspect, white the caudal aspect, red the patient’s right side and yellow the patient’s left side. The white arrow shows an additional orientation mark, placed at the 12 o’clock position using a purple surgical marker to facilitate orientation during imaging.

To ensure accurate orientation, the tissue was marked at the 12 o’clock position using a purple surgical marker and a small incision was made at the same point (Figure 3). This reference point was used to maintain consistent orientation throughout imaging. The tissue was then placed on a prepared sample tray with paraffin oil and secured using customized sponges and a magnetic plate. The tray was inserted along a rail system into the imaging device, positioning the tissue beneath the handheld unit (Figure 2b). Silicone oil was applied through designated injection holes as an optical contact medium.

Tissue navigation and imaging were performed using a joystick controller, always starting at the 12 o’clock incision. Video scans of the tissue margins were captured, typically requiring 2–4 videos depending on the lesion size; each video lasted approximately 40 s. If an area suspicious for BCC was identified at the margin, a subsequent 3D mosaic scan was performed for a more detailed visualization. To confirm the diagnosis and subtype, images were also acquired from representative regions in the center of the specimen.

For the present analysis, imaging was restricted to the four lateral margin quadrants (12–3, 3–6, 6–9, and 9–12 o’clock); the deep margin was not examined systematically and was not included in the primary diagnostic-accuracy analysis.

In selected cases, the specimen was subsequently prepared for evFCM imaging. The lateral margins, defined according to clock-face orientation (12–3, 3–6, 6–9 and 9–12 o’clock), were removed and then stained. Using surgical forceps, the tissue was first rinsed in phosphate-buffered saline (PBS) for 10 s to remove residual blood, followed by immersion in ethanol for 10 s and in the fluorescent dye acridine orange for 30 s. Finally, the tissue was washed again in PBS for 10 s to remove excess dye and was gently dried on a paper towel. The stained tissue was repositioned on a slide using a customized sponge, ensuring that the epidermis was aligned along the longitudinal edge for optimal imaging. The evFCM lens was prepared with a drop of ultrasound gel and imaging was performed for each lateral margin.

After LC-OCT imaging and, in selected cases, evFCM imaging, the specimens were placed in formalin and transferred to the pathology laboratory for processing according to the routine margin-controlled histopathology protocol at each center (Augsburg: Tübinger Torte; Munich: Munich method). LC-OCT was performed immediately after excision and before fixation. No therapeutic intervention occurred between the index test and the reference-standard assessment. Conventional histopathology was selected as the reference standard because it is the established clinical standard for BCC margin assessment. A lateral quadrant was classified as histopathologically positive when BCC was present at the corresponding inked or otherwise identified lateral resection margin. Deep-margin findings were not included in the primary analysis. Histopathologists were blinded to LC-OCT and evFCM findings. Discrepant or uncertain histopathological findings were resolved by consensus (J.W. and E.S.). Histopathological findings were then compared with LC-OCT and, where available, evFCM findings.

LC-OCT datasets were initially assessed by two novice readers (M.M. and K.F.) and two experienced readers (Sa.S. and M.D.), all of whom were blinded to the histopathological results. Histopathological assessment was performed independently by experienced dermatopathologists (J.W. and E.S.). Before evaluating the study images, the readers received LC-OCT image-interpretation training via the DAMAE platform and at the participating study centers and applied predefined ex vivo BCC criteria. During the index-test assessment, readers had access to all available LC-OCT imaging modes, the surface image, dermoscopy, and the AI-generated BCC probability score; no histopathological information was available. Each lateral quadrant was classified as positive, negative, or non-evaluable, and a separate overall lesion-level consensus classification was recorded. Following the individual assessments, discrepant classifications were discussed, and the imaging data were jointly re-evaluated until a single consensus classification was reached. Consensus was reached before disclosure of the histopathological findings. Individual reader-level classifications and formal records of the number, direction and nature of disagreements were not prospectively retained. Consequently, inter-reader agreement and the potential effect of reader experience could not be quantified retrospectively.

The primary analysis evaluated diagnostic performance in the post-training inclusion phase; analysis of the complete cohort was secondary. Lesion-level and quadrant-level LC-OCT classifications were recorded and analyzed separately. At the lesion level, the overall consensus LC-OCT assessment was classified as positive when residual BCC was identified at a lateral margin. A negative overall lesion-level assessment was considered determinate only when all four lateral quadrants were evaluable and negative. If the overall assessment was negative but at least one quadrant was non-evaluable, the lesion-level LC-OCT result was classified as indeterminate and excluded from the primary lesion-level diagnostic-accuracy analysis. The original pragmatic approach, in which these indeterminate results were counted as negative, was retained as a sensitivity analysis. At quadrant level, non-evaluable quadrants were excluded. Histopathological findings were classified as positive at the lesion level when at least one lateral quadrant was positive and as negative at the lesion level when all four quadrants were negative.

Two-by-two tables were used to calculate sensitivity, specificity, accuracy, positive predictive value and negative predictive value, each with 95% confidence intervals. Exact two-sided 95% confidence intervals for lesion-level measures were calculated using the Clopper–Pearson method. For quadrant-level measures, cluster-adjusted 95% confidence intervals were estimated using a nonparametric cluster bootstrap with the lesion as the resampling unit, 1,000,000 bootstrap replications, and a random seed of 20,260,724. All evaluable quadrants from the same lesion were resampled together to account for within-lesion correlation, and percentile-based confidence intervals were reported.

No formal sample-size calculation was performed because this was an exploratory pilot study; the sample size reflected the number of eligible lesions available during the recruitment period. Descriptive statistics for demographic variables were calculated using Microsoft Excel (version 16.110; Microsoft Corporation, Redmond, WA, USA). Diagnostic-accuracy analyses were performed using R (version 4.5.0; R Foundation for Statistical Computing, Vienna, Austria) in RStudio (version 2021.09.0; Posit Software, PBC, Boston, MA, USA). The study was not registered in a public study registry, and the full study protocol is not publicly available.

## 3. Results

Overall, 55 lesions, clinically suspected of being BCC, in 48 patients were examined using ex vivo LC-OCT. Histopathology confirmed BCC in 54 lesions; the remaining lesion showed scar tissue without residual BCC after a previous biopsy. The cohort comprised 20 women and 28 men, with a mean age of 74.83 ± 12.86 years (range, 37–94 years). Of the 55 lesions, 44 cases were recruited at University Hospital Augsburg and 11 at LMU University Hospital Munich.

The lesions were located on the head and neck (*n* = 40, 72.73%), scalp (*n* = 4, 7.27%), trunk (*n* = 9, 16.36%), upper extremities (*n* = 1, 1.82%) and lower extremities (*n* = 1, 1.82%). Measurements were stratified into a training phase and a post-training inclusion phase. Statistical analyses were performed for the complete cohort and separately for the post-training inclusion phase (Table 1). The flow of patients, lesions, and quadrants is shown in Figure 1.

### 3.1. Lesion- and Quadrant-Level Analyses

**Figure 4:**
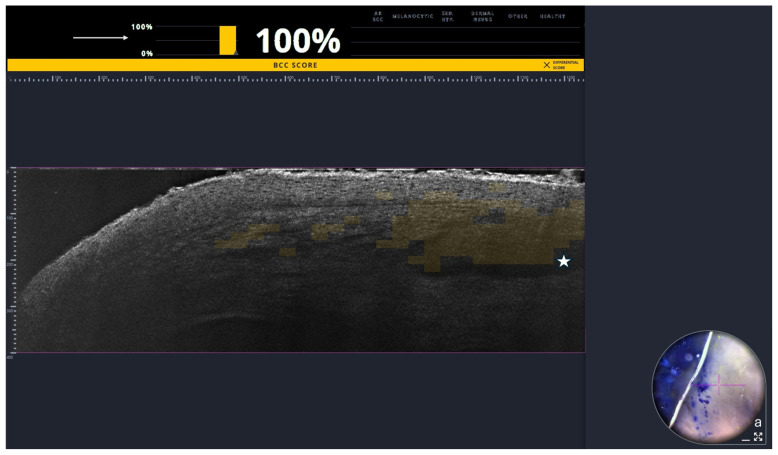
Ex vivo LC-OCT image acquired during margin examination acquired with the deepLive^TM^ system (DAMAE Medical, Paris, France). The white asterisk indicates a basal cell carcinoma (BCC) tumor nest, while the yellow overlay represents the AI-generated heatmap indicating a high probability of BCC. The white arrow points to the AI-generated BCC score of 100% in this example (a). The corresponding dermoscopic images are displayed in the bottom-right corner.

**Figure 5:**
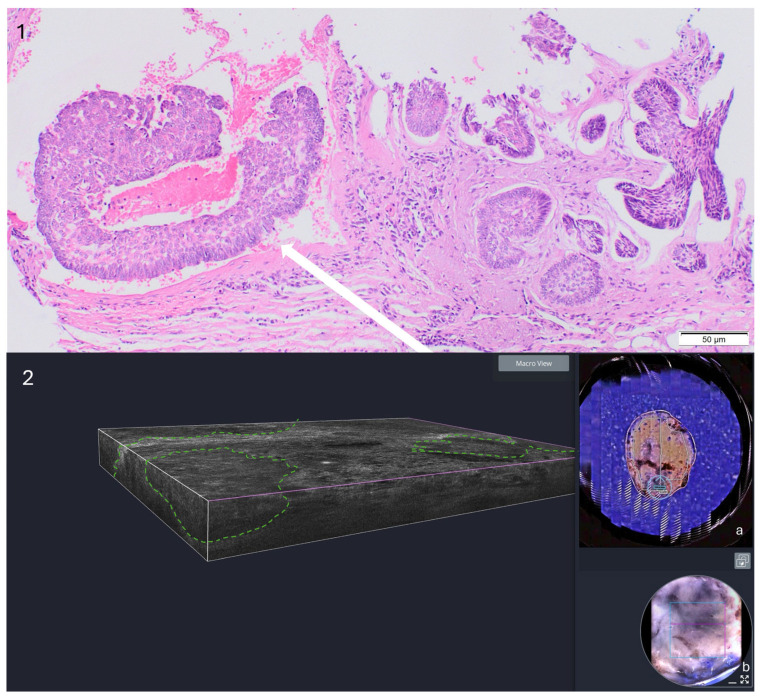
Corresponding histopathological and ex vivo Line-field confocal optical coherence tomography (LC-OCT) findings. Image (**1**): Hematoxylin-and-eosin-stained (H&E) histological section showing a basal cell carcinoma (BCC) tumor nest (white arrow; 10× magnification). Image (**2**): Three-dimensional ex vivo LC-OCT stack showing BCC nests from the same patient (green outline). (**a**) Overview scan of the entire lesion. The blue circle shows the location of the corresponding scan. (**b**) The bottom-right corner shows the corresponding dermoscopic image.

Diagnostic performance was evaluated separately at the lesion level and quadrant levels using histopathology as the reference standard. The lesion-level LC-OCT result was the separately recorded overall consensus assessment of the lateral margins; it was not mechanically derived from the four quadrant labels. Histopathological and LC-OCT findings were classified as positive at the lesion level when residual BCC was identified in at least one lateral-margin quadrant. A lesion was classified as negative at the lesion level only if all four quadrants were evaluable and negative (Figure 4). At the quadrant level, each of the four clock-face quadrants was evaluated individually and compared with the corresponding histopathological quadrant. If a positive margin was suspected, additional images were acquired using the 3D stack mode (Figure 5). The quadrants were defined as 12–3, 3–6, 6–9, and 9–12 o’clock, with 12 o’clock corresponding to the cranial aspect and 6 o’clock to the caudal aspect.

A quadrant was considered concordant when residual tumor was detected within the corresponding angular region (Figure 5). For example, if histopathology identified tumor remnants in the 5–8 o’clock region and ex vivo LC-OCT demonstrated positive margins in the 3–6 and 6–9 o’clock quadrants, both quadrants were considered concordant (Figure 5).

### 3.2. Combined Training and Inclusion Phases

In the complete cohort, histopathology classified 17 lesions as lateral-margin positive and 38 as lateral-margin negative. Two lesions had an overall negative LC-OCT assessment but one or more non-evaluable quadrants and were therefore classified as indeterminate and excluded from the primary lesion-level analysis. The remaining 53 lesions yielded 14 true-positive, 29 true-negative, 3 false-negative, and 7 false-positive results. This corresponded to a sensitivity of 82.35% (95% CI, 56.57–96.20%), specificity of 80.56% (95% CI, 63.98–91.81%), accuracy of 81.13% (95% CI, 68.03–90.56%), positive predictive value of 66.67% (95% CI, 43.03–85.41%) and negative predictive value of 90.63% (95% CI, 74.98–98.02%). At the quadrant level, 214 out of 220 quadrants were evaluable. Six quadrants were non-evaluable because of insufficient image quality or missing imaging data. Among the 214 evaluable quadrants, LC-OCT yielded 32 true-positive, 148 true-negative, 10 false-negative, and 24 false-positive results. Sensitivity was 76.19% (cluster-adjusted 95% CI, 60.47–90.24%), specificity was 86.05% (cluster-adjusted 95% CI, 75.90–94.80%), accuracy was 84.11% (cluster-adjusted 95% CI, 75.60–91.63%), positive predictive value was 57.14% (cluster-adjusted 95% CI, 35.56–80.39%) and negative predictive value was 93.67% (cluster-adjusted 95% CI, 88.36–97.96%) (Table 2).

### 3.3. Post-Training Inclusion Phase

The training phase comprised 23 lesions from 17 patients, and the post-training inclusion phase comprised 32 lesions from 31 patients. Of the 32 inclusion-phase lesions, 3 were recruited in Munich and 29 in Augsburg. No formal sample-size calculation was performed because this was an exploratory pilot study. Three lesions acquired during the nominal inclusion period were assigned to the training phase because they represented the examiners’ first measurements. The inclusion-phase cohort comprised 18 male and 13 female patients, with a mean age of 72.58 ± 12.14 years (range, 37–89 years). At the lesion level, two lesions had an overall negative LC-OCT assessment but at least one non-evaluable quadrant and were classified as indeterminate. Both were histologically lateral-margin negative, but histopathology was not used to determine their LC-OCT classification. Among the 30 determinate lesions, LC-OCT yielded 5 true-positive, 22 true-negative, 2 false-negative, and 1 false-positive results. Sensitivity was 71.43% (95% CI, 29.04–96.33%), specificity was 95.65% (95% CI, 78.05–99.89%) and accuracy was 90.00% (95% CI, 73.47–97.89%). At the quadrant level, 125 out of 128 quadrants were evaluable. LC-OCT yielded 13 true-positive, 106 true-negative, 2 false-negative, and 4 false-positive results, corresponding to a sensitivity of 86.67% (cluster-adjusted 95% CI, 50.00–100.00%), specificity of 96.36% (cluster-adjusted 95% CI, 88.57–100.00%), accuracy of 95.20% (cluster-adjusted 95% CI, 87.30–100.00%), positive predictive value of 76.47% (cluster-adjusted 95% CI, 33.33–100.00%) and negative predictive value of 98.15% (cluster-adjusted 95% CI, 94.12–100.00%).

### 3.4. Descriptive Ex Vivo Fusion Confocal Microscopy Results

In addition to ex vivo LC-OCT, 13 lesions were examined using evFCM for an exploratory descriptive comparison. Ten lesions were available for comparison with LC-OCT. Three were excluded: one because of inadequate evFCM image quality and two because corresponding LC-OCT data were unavailable. In the latter these two lesions, evFCM and histopathology showed concordant findings. Because the cases were selected nonconsecutively and the sample was small, no diagnostic-performance estimates were calculated. The remaining 10 lesions were obtained from five male and five female patients with a mean age of 81.3 years. Lesions were located on the neck (*n* = 1), forehead (*n* = 4), nose (*n* = 4), and cheek (*n* = 1). The findings supported the technical feasibility of multimodal ex vivo assessment but were considered preliminary. In three lesions, eight of twelve quadrants showed concordant negative findings on histopathology, evFCM, and ex vivo LC-OCT. No corresponding ex vivo LC-OCT measurements were available for the remaining four quadrants; evFCM and histopathology were concordantly negative in these regions. In one lesion, only the two quadrants spanning the 6–12 o’clock region were assessed using ex vivo LC-OCT and evFCM. Both evaluated quadrants were positive and concordant with histopathology, which demonstrated tumor involvement in all four quadrants. In another lesion, ex vivo LC-OCT and histopathology were concordant, and the single quadrant additionally examined by evFCM confirmed the positive margin. In two lesions, evFCM and histopathology showed negative margins, whereas ex vivo LC-OCT yielded false-positive findings. Conversely, in one lesion, evFCM yielded a false-positive margin, while histopathology and ex vivo LC-OCT were negative. In one lesion, evFCM and ex vivo LC-OCT showed negative margins, whereas histopathology demonstrated residual infiltrative BCC; this discrepancy may have been related to tumor extension beyond the imaging depth of LC-OCT. Finally, one lesion showed complete agreement among all three modalities, with a positive margin in quadrant 2 and negative findings in the remaining quadrants. Because the index and reference tests were performed on excised tissue, test-related patient adverse events were not applicable.

## 4. Discussion

In this prospective pilot study, ex vivo LC-OCT demonstrated high specificity and overall accuracy for lateral BCC margin assessment in the post-training cohort. After exclusion of two indeterminate results, lesion-level sensitivity was 71.4%, specificity was 95.7%, and accuracy was 90.0%. Sensitivity was based on only seven histologically margin-positive lesions and was therefore imprecise. Quadrant-level performance was encouraging, but the four quadrants were clustered within lesions and should not be interpreted as independent observations. The evFCM substudy was exploratory and too small and selectively sampled to permit for formal comparison. Given the observed false-negative findings and substantial uncertainty around lesion-level sensitivity, ex vivo LC-OCT should not currently replace histopathological margin assessment or serve as the sole basis for definitive wound closure. The present study evaluated diagnostic accuracy but did not assess the safety or clinical outcomes of an LC-OCT guided surgical pathway.

Previous studies have investigated different ex vivo imaging techniques for margin assessment. For instance, a small study by Savant et al. highlighted the potential of perioperative ex vivo dermoscopy, particularly in pigmented BCC [14]. Bennàssar et al. further demonstrated that evFCM may serve as a reliable and time-efficient alternative to frozen sections, reporting a sensitivity of 88% and specificity of 99% for detecting residual tumor [15]. Leemans et al. suggested that bedside ex vivo examination using evFCM may offer potential financial and time-saving advantages for the healthcare system because patients can usually be treated in a single operation without hospitalization [16]. Grizzetti et al. also reported promising results for detecting BCC margins using ex vivo confocal laser scanning microscopy (CLSM), with a sensitivity of 80% and specificity of 100% [17]. These findings are consistent with our own observations during evFCM measurements.

Virtual staining in confocal microscopy is already a widely used technique that, in combination with deep-learning algorithms, may generate hematoxylin-and-eosin-like images that closely resemble conventional histology [18]. By modifying staining protocols and combining reflectance and fluorescence modes in the so-called fusion mode, BCC structures may be differentiated more clearly [19]. In addition, fluorescence confocal mosaicking microscopy has demonstrated high accuracy for detecting residual BCC and further supports the concept of implementing bedside pathology [20]. However, as noted by Longo et al., challenges remain in FCM diagnostics, particularly in distinguishing infiltrative BCC subtypes and differentiating sebaceous glands from BCC tumor nests, which may lead to diagnostic errors [21]. In addition to ex vivo FCM, the literature describes several other imaging techniques for bedside ex vivo margin assessment. Jerjes et al., for example, evaluated the use of OCT immediately after excision of cutaneous lesions and, despite the limited number of cases, reported promising diagnostic accuracy, particularly for actinic keratosis and BCC [22]. Similarly, Rashed et al. compared ex vivo OCT assessment of BCC margins with conventional histology and reported good diagnostic agreement [23]. Other studies have suggested that although ex vivo OCT enables the identification of BCC, limitations including suboptimal image quality and challenges in margin detection may restrict its performance compared with frozen-section histology [24,25].

Jain et al. applied high-resolution full-field OCT to freshly excised BCC specimens and demonstrated that this method can facilitate surgical margin assessment, particularly when supported by artificial intelligence (AI) [26]. Furthermore, Iftimia et al. showed that combining RCM and OCT measurements in a single device may enhance margin assessment by providing complementary information in the ex vivo evaluation of BCC [27]. The primary focus of the present study was the application of ex vivo LC-OCT for margin assessment of excised BCC specimens. Although LC-OCT has already demonstrated high diagnostic performance for in vivo BCC diagnosis, its application in the ex vivo setting has not yet been thoroughly investigated [28,29,30]. Suppa et al. demonstrated that LC-OCT improves diagnostic accuracy for BCC when used in vivo [30], and Jacobsen et al. showed that LC-OCT can support precise and tissue-sparing excisions through targeted in vivo margin mapping [31]. Furthermore, Cinotti et al. demonstrated that as little as one hour of LC-OCT training may improve the diagnostic accuracy of beginners in the in vivo setting, suggesting that the device is relatively easy to learn [32]. The technical feasibility of integrating ex vivo LC-OCT into a combined in vivo–ex vivo BCC workflow was previously described by our group in the methodological “BCC-One-Stop-Shop” publication [9]. That report focused on workflow development, co-localized imaging, AI-supported visualization, and standardized operating procedures and illustrated the approach using two representative clinical cases. It did not provide cohort-level diagnostic-performance analyses. The present study extends that methodological work by providing the previously announced prospective, blinded, two-center evaluation of ex vivo LC-OCT against conventional histopathology. Its added contribution lies in the comprehensive lesion- and quadrant-level diagnostic analysis, the separate evaluation of the training and inclusion phases, the explicit consideration of non-evaluable quadrants, and the exploratory descriptive comparison with evFCM. Accordingly, the novelty of the present study lies in the systematic diagnostic-performance evaluation of ex vivo LC-OCT rather than in the initial introduction of the ex vivo workflow itself. Ogien et al. previously noted that diagnostic criteria established for in vivo LC-OCT imaging may also be applied to ex vivo imaging [6], which is consistent with our observations. Moreover, as in the in vivo setting, the LC-OCT software includes an AI-based BCC probability score that may support less experienced users in clinical practice [9]. Fischman et al. demonstrated that AI-assisted evaluation may enhance in vivo diagnosis of BCC using LC-OCT, particularly among less experienced users [12]. However, the role of AI support in ex vivo LC-OCT margin assessment remains to be systematically evaluated.

Despite these advantages, several challenges are specific to the ex vivo application of LC-OCT. A key source of potential error is the positioning of the excised tissue within the sample holder. Pressure applied during mounting may alter tissue depth and structure, which can lead to misinterpretation of tumor margins. This issue has also been described by Ogien et al., who noted that excessive pressure can distort the tissue, and the natural curvature of the specimen may prevent imaging of the full margin depth [6]. Similar observations were made in our study.

In thicker specimens, excessive compression occasionally caused deep subcutaneous fat to appear in contact with the imaging plate, thereby creating an artificial margin. Although this compression may allow visualization of deeper structures, such findings must be interpreted with caution to avoid misjudging the true tumor boundary. We aimed to consistently image the true surgical margins, as identified in the dermatoscopic overview image and inferred from epithelial structures in the ex vivo LC-OCT images. Nevertheless, we cannot confirm definitively that the correct margin was captured in all cases. Another practical limitation is the size of the imaging tray (3 cm in diameter), which restricts the maximum lesion size that can be evaluated using ex vivo LC-OCT. One potential approach to overcome this limitation would be to section larger BCCs into smaller specimens before imaging; however, this strategy requires further investigation and must not compromise subsequent histopathological assessment. Tissue orientation is another practical challenge. Accurate orientation is essential for correlating imaging findings with histology and for guiding potential re-excisions. To ensure consistent orientation, the 12 o’clock position of each specimen was marked with a scalpel incision and a purple surgical marker. This reference point was clearly visible in both the LC-OCT images and in the corresponding surface photographs, allowing more precise localization of tumor-positive margins during analysis.

An additional advantage of the method is the possibility of inverting the specimen to examine the deep margin for residual tumor. However, this approach was not systematically incorporated into the study protocol and was not applied consistently. Ex vivo LC-OCT also requires less specimen preparation than evFCM because no staining protocol is needed. Moreover, LC-OCT can be used in both in vivo and ex vivo modes on a single imaging platform, which may facilitate integrated diagnostic and surgical workflows. The present study did not perform a formal cost analysis, and no cost-saving claim can be made from these data.

Beyond ex vivo assessment of the excised specimen, direct in vivo LC-OCT imaging of open-wound margins could be explored as a complementary approach. In principle, this could support immediate assessment of suspicious wound-edge areas and more targeted, tissue-sparing re-excision. However, open-wound LC-OCT imaging was not evaluated in the present study. Feasibility studies would need to establish a validated sterile interface, atraumatic and reproducible contact with the wound surface, adequate optical coupling and image quality on irregular or bleeding tissue, and reliable spatial co-registration with subsequent re-excision and histopathological assessment.

If validated prospectively, rapid ex vivo margin assessment could potentially shorten the interval to wound closure and reduce staged procedures. However, the present study did not evaluate the complete clinical pathway or determine whether the use of ex vivo LC-OCT reduces the number of surgical stages, operative time, hospitalization, costs, complications, or patient-reported outcomes. These potential workflow-related, clinical, and health-economic effects require dedicated prospective evaluation before an LC-OCT-based one-stop-shop approach, encompassing the entire diagnostic and therapeutic workflow, can be recommended. Nevertheless, this concept is a relevant topic for future research because it may offer several advantages. A more streamlined intraoperative workflow and improved procedural efficiency could reduce operative time [16]. Immediate wound closure may also lower the risk of complications, shorten hospital stays, and potentially reduce costs [3,16]. In addition, combining diagnosis and treatment within a single procedure may improve patient satisfaction [33].

Several limitations must be considered. First, the small sample size limits generalizability. Second, the lesions formed a nonconsecutive convenience series selected during surgical consultations, partly in the context of a parallel in vivo imaging study. This may have favored smaller or more clearly demarcated BCCs, for example, lesions with less ulceration, and may have overestimated diagnostic performance. Third, spectrum bias may be present, if certain histological subtypes or more complex tumor morphologies were underrepresented. Ulceration and tissue disruption after excision may also impair ex vivo LC-OCT image quality because irregular surfaces can introduce artifacts and hinder optimal contact with the imaging plate.

Fourth, although a training phase was defined, a residual learning effect during the inclusion phase cannot be excluded, potentially influencing diagnostic performance. Fifth, although histopathology was used as the reference standard, sampling limitations inherent to histological sectioning at both centers may have affected the comparison. Accurate spatial correlation between LC-OCT findings and histopathology remains challenging. Despite the use of a clock-face orientation, tissue deformation during excision and processing may have led to partial misalignment, potentially affecting quadrant-level agreement.

Additional limitations include the small number of histologically margin-positive lesions, which resulted in imprecise sensitivity estimates; the non-independence of quadrants within lesions and of multiple lesions within some patients; and the absence of a prespecified patient-level clustering analysis. Two lesions had an overall negative LC-OCT assessment but one or more non-evaluable quadrants. They were classified as indeterminate and excluded from the primary lesion-level analysis; the original pragmatic classification is reported only as a sensitivity analysis. Six quadrants were non-evaluable in the complete cohort, emphasizing the need for prospectively defined acquisition-quality and evaluability criteria. Individual reader-level classifications and formal records of disagreements were not retained; consequently, disagreement frequency, inter-reader agreement, and the influence of reader experience could not be reconstructed. The reported estimates are based on consensus classifications and do not quantify reproducibility. In addition, the AI score was developed for in vivo imaging and was not independently validated for ex vivo margin assessment. Finally, deep-margin assessment was not systematically incorporated into the study protocol. Future studies should use standardized acquisition, prospectively retained reader-level assessments, predefined consensus procedures, systematic deep-margin evaluation, rigorous histopathological co-registration, and larger consecutively recruited cohorts.

## 5. Conclusions

Ex vivo LC-OCT demonstrated promising diagnostic performance for lateral BCC margin assessment in this pilot cohort, with particularly high specificity after the training phase. However, the limited number of margin-positive lesions, nonconsecutive selection, indeterminate results, non-independent quadrant observations, incomplete evaluability, and absence of systematic deep-margin assessment preclude conclusions regarding clinical effectiveness or the replacement of histopathology. Histopathological verification remains required. Larger prospective studies using standardized acquisition, prospectively documented reader assessments, predefined consensus procedures, cluster-aware statistical analysis, rigorous histopathological co-registration and systematic deep-margin imaging are required before routine clinical implementation.

## Figures and Tables

**Table 1:** Demographic and clinical characteristics of the ex vivo LC-OCT cohort.

Variables		Complete Cohort (Training + Post-Training Inclusion)	Post-Training Inclusion Phase
Patients		48	31
Gender *n* (%)	female	20 (41.67%)	13 (41.94%)
	male	28 (58.33%)	18 (58.06%)
Age (mean)		74.83 ± 12.86 years (range, 37–94 years)	72.58 ± 12.14 years (range, 37–89 years)
Recruiting center–patients *n* (%)	Munich Augsburg	8 (16.67%) 40 (83.33%)	3 (9.68%) 28 (90.32%)
Lesions		55	32
Quadrants	All Evaluable	220 214	128 125
Histopathological diagnosis/subtypes	Superficial Nodular Infiltrative No residual BCC/scar tissue only	8 (14.55%) 24 (43.64%) 22 (40.00%) 1 (1.82%)	5 (15.63%) 17 (53.13%) 10 (31.25%) 0 (0%)
Localization *n* (%)	Head and neck Scalp Trunk Upper extremities Lower extremities	40 (72.73%) 4 (7.27%) 9 (16.36%) 1 (1.82%) 1 (1.82%)	25 (78.13%) 3 (9.38%) 3 (9.38%) 1 (3.13%) 0 (0%)
Recruiting center–lesions *n* (%)	Munich	11 (20%)	3 (9.38%)
	Augsburg	44 (80%)	29 (90.63%)

**Table 2:** Diagnostic performance of ex vivo LC-OCT compared with histopathology.

Measure		Complete Cohort (Training + Post-Training Inclusion)	Post-Training Inclusion Phase
**Lesion-level analysis**	Analyzed lesions	55	32
Histopathology	Indeterminate results excluded, *n*	2	2
	True positive (TP), *n*	14	5
	True negative (TN), *n*	29	22
	False negative (FN), *n*	3	2
	False positive (FP), *n*	7	1
	Sensitivity, % (95% CI)	82.35 (56.57–96.20)	71.43 (29.04–96.33)
	Specificity, % (95% CI)	80.56 (63.98–91.81)	95.65 (78.05–99.89)
	Accuracy, % (95% CI)	81.13 (68.03–90.56)	90.00 (73.47–97.89)
	Positive predictive value, % (95% CI)	66.67 (43.03–85.41)	83.33 (35.88–99.58)
	Negative predictive value, % (95% CI)	90.63 (74.98–98.02)	91.67 (73.00–98.97)
**Quadrant-level analysis**	Analyzed quadrants	220	128
Histology	Non-evaluable excluded, *n*	6	3
	True positive (TP), *n*	32	13
	True negative (TN), *n*	148	106
	False negative (FN), *n*	10	2
	False positive (FP), *n*	24	4
	Sensitivity, % (cluster-adjusted 95% CI)	76.19 (60.47–90.24)	86.67 (50.00–100.00)
	Specificity, % (cluster-adjusted 95% CI)	86.05 (75.90–94.80)	96.36 (88.57–100.00)
	Accuracy, % (cluster-adjusted 95% CI)	84.11 (75.60–91.63)	95.20 (87.30–100.00)
	Positive predictive value, % (cluster-adjusted 95% CI)	57.14 (35.56–80.39)	76.47 (33.33–100.00)
	Negative predictive value, % (cluster-adjusted 95% CI)	93.67 (88.36–97.96)	98.15 (94.12–100.00)

## Data Availability

The de-identified lesion-level and quadrant-level datasets underlying the reported diagnostic-accuracy estimates, together with the corresponding R analysis code, data dictionary, and README, are provided as Supplementary Materials. The complete raw LC-OCT imaging dataset is not publicly available because of its volume and the presence of embedded examination-linked metadata. Subject to institutional, ethical, and data-protection requirements, selected de-identified representative LC-OCT image and video data may be obtained from the corresponding author upon reasonable request. The methodological relationship between the present study and the previously published “BCC-One-Stop-Shop” workflow article is described in the Introduction and Methods. No cohort-level diagnostic-performance analyses reported in the present manuscript were published previously.

## Supplementary Materials

The following supporting information can be downloaded at: https://www.mdpi.com/article/10.3390/cancers18152509/s1, Table S1, completed STARD 2015 checklist; Table S2, sensitivity analysis of alternative handling of indeterminate lesion-level results; Table S3, quadrant-level LC-OCT and histology results for TP, TN, FP, and FN examples of Figures S4–S7. Figures S4–S7 For independent diagnosis of BCC four representative cases with LC-OCT images from all four quadrants of each case are provided in TIFF format. All images have been stripped of personal and patient-identifying information. Data S1, de-identified lesion-level and quadrant-level analysis workbook with a data dictionary and README; Code S1, R analysis script provided to reproduce the diagnostic-performance estimates and confidence intervals.

## Abbreviations

The following abbreviations are used in this manuscript:

BCC	Basal cell carcinoma
evFCM	Ex vivo fusion confocal microscopy
LC-OCT	Line-field confocal optical coherence tomography
OCT	Optical coherence tomography
RCM	Reflectance confocal microscopy
AI	Artificial intelligence
FOV	Field of view
