# Supplementary material for: Ex Vivo Line-Field Confocal Optical Coherence Tomography and Ex Vivo Fusion Confocal Microscopy for Lateral-Margin Assessment of Basal Cell Carcinoma in Correlation with Histopathology: A Prospective Pilot Diagnostic-Accuracy Study
Source: Cancers (Basel). 2026 Aug 5;18(15):2509. doi: 10.3390/cancers18152509 (PMC13463871; doi:10.3390/cancers18152509)
Supplement: Supplementary file 1 [file cancers-18-02509-s001.zip › Supplementary_Tables_S1_S2_S3.pdf]

Supplementary Tables

Supplementary Table S1. STARD 2015 checklist for the revised manuscript

The checklist follows the STARD 2015 reporting guideline for diagnostic-accuracy studies.

Section and topic	Item	STARD 2015 checklist item	Reported on page / location
TITLE OR ABSTRACT	1	Identification as a study of diagnostic accuracy using at least one measure of accuracy (such as sensitivity, specificity, predictive values, or AUC)	p. 1-2 (title and Abstract)
ABSTRACT	2	Structured summary of study design, methods, results, and conclusions	p. 1-2 (Abstract)
INTRODUCTION	3	Scientific and clinical background, including the intended use and clinical role of the index test	p. 2-3 (Introduction)
INTRODUCTION	4	Study objectives and hypotheses	p. 3 (end of Introduction)
METHODS—Study design	5	Whether data collection was planned before the index test and reference standard were performed (prospective study) or after (retrospective study)	pp. 3-5 (Materials and Methods opening)
METHODS—Participants	6	Eligibility criteria	p. 5(Section 2.1)
METHODS—Participants	7	On what basis potentially eligible participants were identified (such as symptoms, results from previous tests, inclusion in registry)	p. 5 (Section 2.1)
METHODS—Participants	8	Where and when potentially eligible participants were identified (setting, location, and dates)	p. 5 (Section 2.1)
METHODS—Participants	9	Whether participants formed a consecutive, random, or convenience series	p. 5 (Section 2.1; convenience, non-consecutive)
METHODS—Test methods	10a	Index test, in sufficient detail to allow replication	pp. 5-9 (Sections 2.2 and 2.4)
METHODS—Test methods	10b	Reference standard, in sufficient detail to allow replication	p. 9 (Section 2.4)
METHODS—Test methods	11	Rationale for choosing the reference standard (if alternatives exist)	p. 9 (Section 2.4)
METHODS—Test methods	12a	Definition of and rationale for test positivity cut-offs or result categories of the index test, distinguishing prespecified from exploratory	p. 8-9 (Section 2.4; positive, negative, non-evaluable/indeterminate)
METHODS—Test methods	12b	Definition of and rationale for test positivity cut-offs or result categories of the reference standard, distinguishing prespecified from exploratory	p. 8-9 (Section 2.4)
METHODS—Test methods	13a	Whether clinical information and reference-standard results were available to the performers/readers of the index test	p. 7-9 (reader-assessment paragraph)
METHODS—Test methods	13b	Whether clinical information and index-test results were available to the assessors of the reference standard	p. 8-9 (histopathologists blinded)
METHODS—Analysis	14	Methods for estimating or comparing measures of diagnostic accuracy	p. 9 (statistical-analysis paragraph)
METHODS—Analysis	15	How indeterminate index-test or reference-standard results were handled	p. 9 (classification paragraph); Table S2
METHODS—Analysis	16	How missing data on the index test and reference standard were handled	p. 9 (non-evaluable quadrants excluded; histopathology complete)
METHODS—Analysis	17	Any analyses of variability in diagnostic accuracy, distinguishing prespecified from exploratory	pp. 3-5 and 7-9; Results pp. 12-14 (primary post-training and secondary complete-cohort analyses)
METHODS—Analysis	18	Intended sample size and how it was determined	p. 3,5,9 (pilot study; no formal calculation)
RESULTS—Participants	19	Flow of participants, using a diagram	p. 4 (Figure 3)
RESULTS—Participants	20	Baseline demographic and clinical characteristics of participants	p. 10 (Table 1)
RESULTS—Participants	21a	Distribution of severity of disease in those with the target condition	p. 10 (Table 1; histopathological BCC subtypes)
RESULTS—Participants	21b	Distribution of alternative diagnoses in those without the target condition	p. 10 (Table 1). The target condition was residual BCC at the lateral margin; one scar-only lesion is reported.
RESULTS—Participants	22	Time interval and any clinical interventions between index test and reference standard	pp. 7-8 (immediate LC-OCT before fixation; no therapeutic intervention)
RESULTS—Test results	23	Cross-tabulation of index-test results (or their distribution) by reference-standard results	p. 13 (Table 2)
RESULTS—Test results	24	Estimates of diagnostic accuracy and their precision (such as 95% confidence intervals)	pp. 12-14 (Sections 3.2–3.3 and Table 2)
RESULTS—Test results	25	Any adverse events from performing the index test or reference standard	p. 14 (not applicable at patient level because both tests were performed on excised tissue)
DISCUSSION	26	Study limitations, including sources of potential bias, statistical uncertainty, and generalisability	pp. 15-17 (Discussion)
DISCUSSION	27	Implications for practice, including the intended use and clinical role of the index test	pp. 14-18 (Discussion and Conclusions)
OTHER INFORMATION	28	Registration number and name of registry	p. 9 (study not registered)
OTHER INFORMATION	29	Where the full study protocol can be accessed	p. 9 (protocol not publicly available)
OTHER INFORMATION	30	Sources of funding and other support; role of funders	pp. 18-19 (Funding, Acknowledgments, and Conflicts of Interest)

Supplementary Table S2. Sensitivity analysis of alternative handling of indeterminate lesion-level LC-OCT results

The primary rule excluded lesions with an overall negative LC-OCT assessment and at least one non-evaluable quadrant. The pragmatic sensitivity analysis counted these lesions as LC-OCT negative. Both indeterminate lesions occurred in the post-training inclusion phase and were histologically lateral-margin negative; histopathology was not used to define their LC-OCT classification.

Measure	Complete cohort—primary rule	Complete cohort—pragmatic rule	Inclusion phase—primary rule	Inclusion phase—pragmatic rule
Lesions analyzed, n	55	55	32	32
Indeterminate lesions excluded, n	2	0	2	0
True positive, n	14	14	5	5
True negative, n	29	31	22	24
False negative, n	3	3	2	2
False positive, n	7	7	1	1
Sensitivity, % (95% CI)	82.35 (56.57–96.20)	82.35 (56.57–96.20)	71.43 (29.04–96.33)	71.43 (29.04–96.33)
Specificity, % (95% CI)	80.56 (63.98–91.81)	81.58 (65.67–92.26)	95.65 (78.05–99.89)	96.00 (79.65–99.90)
Accuracy, % (95% CI)	81.13 (68.03–90.56)	81.82 (69.10–90.92)	90.00 (73.47–97.89)	90.63 (74.98–98.02)
Positive predictive value, % (95% CI)	66.67 (43.03–85.41)	66.67 (43.03–85.41)	83.33 (35.88–99.58)	83.33 (35.88–99.58)
Negative predictive value, % (95% CI)	90.63 (74.98–98.02)	91.18 (76.32–98.14)	91.67 (73.00–98.97)	92.31 (74.87–99.05)

Abbreviations: CI, confidence interval; LC-OCT, line-field confocal optical coherence tomography. Exact two-sided Clopper–Pearson confidence intervals are shown.

Supplementary Table S3. Quadrant-level LC-OCT and histology results for TP, TN, FP, and FN examples

Quadrant-level LC-OCT and histology assessments are shown for one example lesion in each result category.

Analysis patient ID	Analysis lesion ID	Center	Phase	Quadrant	LC-OCT quadrant result	Histology quadrant result	Evaluable	Result
P021	L026	Augsburg	inclusion	Q1 (12-3 o'clock)	negative	negative	Yes	TP
P021	L026	Augsburg	inclusion	Q2 (3-6 o'clock)	positive	positive	Yes	TP
P021	L026	Augsburg	inclusion	Q3 (6-9 o'clock)	positive	positive	Yes	TP
P021	L026	Augsburg	inclusion	Q4 (9-12 o'clock)	negative	negative	Yes	TP
P042	L048	Augsburg	inclusion	Q1 (12-3 o'clock)	negative	negative	Yes	TN
P042	L048	Augsburg	inclusion	Q2 (3-6 o'clock)	negative	negative	Yes	TN
P042	L048	Augsburg	inclusion	Q3 (6-9 o'clock)	negative	negative	Yes	TN
P042	L048	Augsburg	inclusion	Q4 (9-12 o'clock)	negative	negative	Yes	TN
P035	L041	Augsburg	inclusion	Q1 (12-3 o'clock)	positive	negative	Yes	FP
P035	L041	Augsburg	inclusion	Q2 (3-6 o'clock)	positive	negative	Yes	FP
P035	L041	Augsburg	inclusion	Q3 (6-9 o'clock)	positive	negative	Yes	FP
P035	L041	Augsburg	inclusion	Q4 (9-12 o'clock)	positive	negative	Yes	FP
P034	L040	Augsburg	inclusion	Q1 (12-3 o'clock)	negative	positive	Yes	FN
P034	L040	Augsburg	inclusion	Q2 (3-6 o'clock)	negative	positive	Yes	FN
P034	L040	Augsburg	inclusion	Q3 (6-9 o'clock)	negative	negative	Yes	FN
P034	L040	Augsburg	inclusion	Q4 (9-12 o'clock)	negative	negative	Yes	FN

Abbreviations: FN, false negative; FP, false positive; LC-OCT, line-field confocal optical coherence tomography; TN, true negative; TP, true positive.
